# Network Analysis of miRNA and Cytokine Landscape in Human Hematopoiesis

**DOI:** 10.3390/ijms252212305

**Published:** 2024-11-16

**Authors:** Alessandro Vici, Germana Castelli, Federica Francescangeli, Annamaria Cerio, Elvira Pelosi, Maria Screnci, Stefania Rossi, Ornella Morsilli, Nadia Felli, Luca Pasquini, Giuseppina Ivana Truglio, Maria Laura De Angelis, Vito D’Andrea, Rachele Rossi, Paola Verachi, Frenki Vila, Giovanna Marziali, Alessandro Giuliani, Ann Zeuner

**Affiliations:** 1 Department of Oncology and Molecular Medicine, Istituto Superiore di Sanità, Viale Regina Elena 299, 00161 Rome, Italy; alessandro.vici@iss.it (A.V.); germana.castelli@iss.it (G.C.); federica.francescangeli@iss.it (F.F.); annamaria.cerio@iss.it (A.C.); elvira.pelosi@iss.it (E.P.); stefania.rossi@iss.it (S.R.); nadia.felli@iss.it (N.F.); marialaura.deangelis@iss.it (M.L.D.A.); rachele.rossi@iss.it (R.R.); paola.verachi@iss.it (P.V.); frenkivila06@gmail.com (F.V.); giovanna.marziali@iss.it (G.M.); 2 Banca Regionale Sangue Cordone Ombelicale, UOC Immunoematologia e Medicina Trasfusionale, Policlinico Umberto I, 00161 Rome, Italy; m.screnci@policlinicoumberto1.it; 3 Department of Cardiovascular, Endocrine-Metabolic Diseases and Ageing, Istituto Superiore di Sanità, 00161 Rome, Italy; ornella.morsilli@iss.it; 4 Core Facilities, Istituto Superiore di Sanità, 00161 Rome, Italy; luca.pasquini@iss.it; 5 Department of Biotechnology, Chemistry and Pharmacy, University of Siena, 53100 Siena, Italy; giuseppina.truglio@unisi.it; 6 Department of Surgery, Sapienza University of Rome, Viale Regina Elena 324, 00161 Rome, Italy; vito.dandrea@uniroma1.it; 7 Environment and Health Department, Istituto Superiore di Sanità, Viale Regina Elena 299, 00161 Rome, Italy

**Keywords:** hematopoiesis, regulatory networks, microRNAs, cytokines, differentiation

## Abstract

The differentiation/maturation trajectories of different blood cell types stemming from a CD34^+^ common ancestor takes place in different biologically relevant multidimensional spaces. Here, we generated microRNA and cytokine profiles from highly purified populations of hematopoietic progenitors/precursors derived from cord blood hematopoietic stem/progenitor cells. MicroRNA and cytokine landscapes were then analyzed to find their mutual relationships under the hypothesis that the highly variable miRNome corresponds to the ‘force field’ driving the goal of a stable phenotype (here corresponding to the cytokine abundance pattern) typical of each cell kind. The high dimensionality and lack of linearity of the hematopoietic process pushed us to adopt a distance–geometry approach to compare different trajectories, while a complex network analysis was instrumental in revealing the fine structure of microRNA–cytokine relations. Importantly, the approach enabled us to identify a limited number of factors (represented either by microRNAs or cytokines) corresponding to crucial nodes responsible for connecting distinct interaction modules. Subtle changes in ‘master nodes’, keeping the connections between different regulatory networks, may therefore be crucial in influencing hematopoietic differentiation. These findings highlight the extremely interconnected network structures underlying hematopoiesis regulation and identify key factors in the microRNA/cytokine landscape that may be potentially crucial for influencing network stability.

## 1. Introduction

Hematopoiesis is the continuous process by which blood cells are produced, maintained at normal levels, and increased in response to demand. Blood cells derive from a small group of pluripotent hematopoietic stem cells (HSCs) that generate progenitors committed to one or a few hematopoietic lineages. This process consists of a network of highly interconnected steps, including, as follows: the generation of stem cells; the maintenance of HSCs by self-renewal; the proliferation and maintenance of multi-potent progenitors; and the lineage commitment and maturation of hematopoietic precursors. Hematopoiesis is a dynamic and adaptable process of blood cell generation in the bone marrow, which can be modulated by a variety of conditions, including infection, trauma, and disease. The various immune cells generated by hematopoiesis can modulate blood cell generation through a variety of effector mechanisms, including the production of cytokines and interferons. Certain immune cells, including neutrophils, macrophages, dendritic cells, B cells, CD4^+^ T cells, CD8^+^ T cells, and Treg cells, establish enduring residencies within the bone marrow. Their distinctive interactions with bone marrow sites of hematopoiesis and with the bone marrow microenvironment are now identified, as well as their multifaceted immune functions. These cells play a dual role in shaping hematopoiesis through direct modulation of the quiescence, self-renewal, and multi-lineage differentiation of hematopoietic stem and progenitor cells via direct cell-to-cell interactions or the secretion of various factors known for their immunological functions or thorough interactions with the cellular constituents of the bone marrow niche, particularly mesenchymal stem cells, endothelial cells, osteoblasts, and osteoclasts, promoting their survival and contributing to tissue repair, thus maintaining a supportive environment for hematopoietic stem and progenitor cells.

The microRNA (miRNA) expression profile is a relevant signature of the hematopoiesis process [1] and has been demonstrated to regulate both HSC self-renewal and the fine-tuning of cell fates during the lineage-specification process [2,3]. MiRNAs are small non-coding RNAs that regulate gene expression by targeting the 3′ untranslated region (UTR) of mRNAs [4]. Physical interactions between miRNAs and their target mRNA induce cleavage of the mRNAs or block the translation of the mRNA into protein, regulating the abundance of different proteins [5,6]. Here, we focus on the differentiation trajectories of blood cell lineages in both miRNA and cytokine concentration profile spaces. Adopting a physical metaphor, the miRNA space corresponds to a time-varying ‘force field’ driving hematopoiesis, while the cytokine pattern is the state-space of the system changing under the action of such a force field.

By using a serum-free culture systems developed for the unilineage differentiation and maturation of cord blood CD34^+^ HSCs through the erythroid (E), monocytic (Mo), granulocytic (G) or megakaryocytic (Mk) pathways, our group demonstrated the tenability of such a metaphor [7], by analyzing the expression profile of protein coding genes (state-space), and miRNAs at sequential stages of development. We observed a coordinated, fully interconnected and scalable character of cell population behavior in both the transcriptome and miRNome reminiscent of an attractor-like dynamics. However, while the transcriptome reached a clear equilibrium state at the end of the process, reminiscent of the attainment of a stable final maturation state, the miRNA space, consistent with its force-field character, did not reach such a ‘terminal state’. This result mirrors the need for a continuous regulation activity to sustain the homeostasis of the cell lineage phenotype. The need for a relentless activity of non-coding RNAs in maintaining the ‘correct’ attractor state was recently demonstrated [8] to be a general feature of biological regulation.

In this work, we investigated, in depth, the relation between ‘regulation’ and ‘phenotype’ views of differentiation and maturation trajectories by the analysis of miRNA and cytokine spaces relative to E, Mo, MK and G lineages. We analyzed these trajectories in terms of departure (expressed as log2 fold change (logFC) for each miRNA species) from their common CD34^+^ ancestor, adopting a non-linear distance-based approach [9]. Both miRNA and cytokine spaces were able to track the specificities of the different lineages’ trajectories with cytokines, consistent with the nature of the phenotypic space, having a more stable and line-specific characterization. We investigated the mutual relations between miRNA and cytokine spaces at the fine grain of molecular species by a complex network approach, revealing lineage-specific regulation architectures.

## 2. Results

To generate step-by-step miRNA and cytokine profiles of differentiating hematopoietic cells, we used a serum-free culture system allowing for the analysis of sequential stages of differentiation/maturation [7]. To this aim, we purified HSCs from donors’ cord blood by positive selection based on CD34 antigen expression. Cells were then cultured in serum-free media, allowing for the selective growth of erythroid, megakaryocytic, granulocytic, and monocytic progenitors/precursors. This selective growth was reached by the agency of specific growth factor combinations, including, as follows: Epo; IL-3; GM-CSF for erythroid cells; Tpo for megakaryocytes; IL-3, GM-CSF, and G-CSF for granulocytes; and IL-6, Flt-3, and M-CSF for monocytes [10,11].

**Figure 1:**
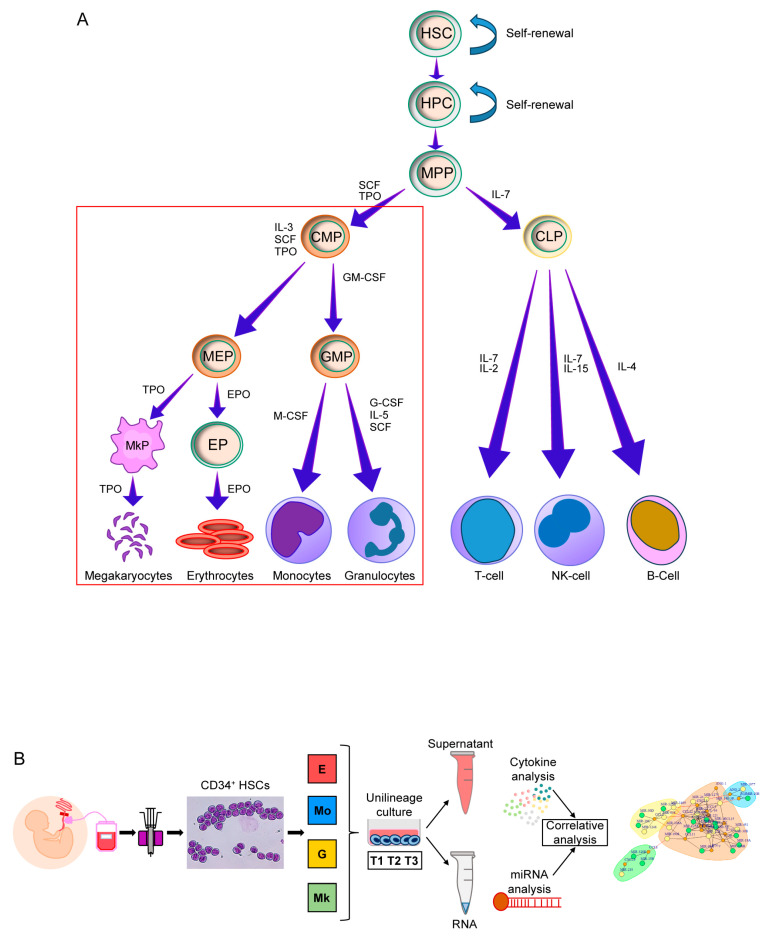
(**A**) The process of hematopoietic differentiation: the red frame highlights myeloid differentiation, which is the object of the analyses performed within this study. Abbreviations are available in in the Supplementary Information. (**B**) Workflow of the study: cord blood (CB) is extracted from umbilical cord and CD34^+^ HSCs are isolated by positive selection. HSCs are cultured with appropriate growth factor combinations in a serum-free medium to obtain pure populations of hematopoietic progenitors/precursors belonging to the E, Mo, G and MK lineages. RNA and culture medium supernatant are collected at three time points (days 5-10-15) from each culture to produce miRNA and cytokine profiles that in turn are the objects of data analysis.

Figure 1 summarizes the hematopoiesis process (Figure 1A) and the workflow of experiments that, starting with the cord blood HSCs purification, led to the generation of miRNA/cytokine networks (Figure 1B).

**Figure 2:**
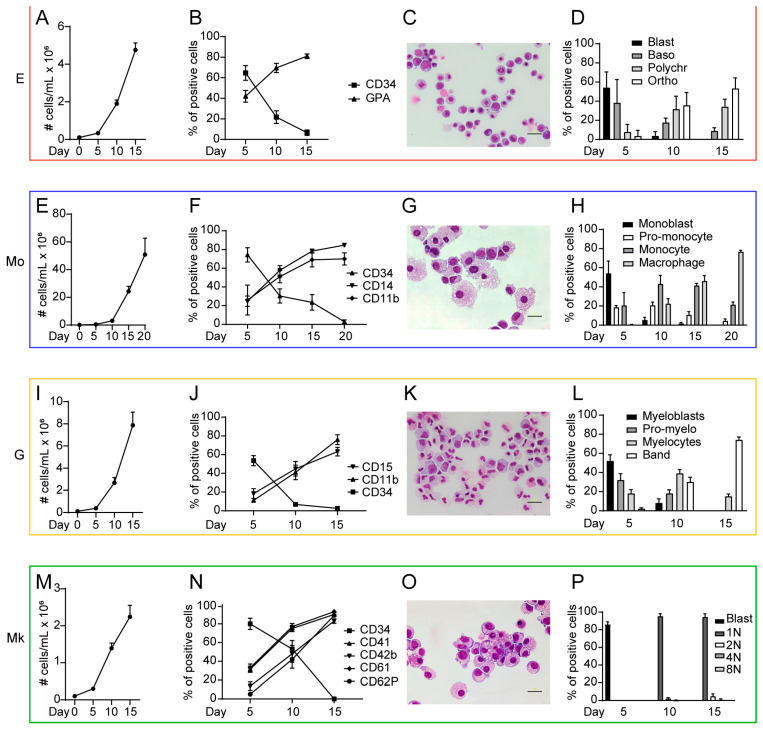
**Panel 1.** Growth and differentiation of E unilineage culture: (**A**) cell growth analysis of E unilineage culture generated by CB CD34^+^ HSCs; (**B**) percentage of CD34- and GPA-positive cells in unilineage erythroid differentiation and maturation; (**C**) representative picture of E at day 15 stained with May–Grünwald Giemsa; (**D**) the graph represents the morphological analysis of E lineage elements at various stages of maturation; and (**A**,**B**,**D**) mean ± SEM of three independent experiments is shown. **Panel 2**. Growth and differentiation of Mo unilineage culture: (**E**) cell growth analysis of Mo unilineage culture generated by CB CD34^+^ HSCs; (**F**) flow cytometry analysis of CD34, CD14 and CD11b expression during Mo differentiation; (**G**) representative picture of Mo at day 15, stained with May–Grünwald Giemsa, is shown; (**H**) the graph represents the morphological analysis of Mo lineage elements at various stages of maturation; and (**E**,**F**,**H**) mean ± SEM of three independent experiments is shown. **Panel 3**. Growth and differentiation of G unilineage culture: (**I**) cell growth analysis of G unilineage culture generated by CB CD34^+^ HSCs; (**J**) Flow cytometry analysis of CD15, CD11b, and CD34 expression during granulocytic differentiation; (**K**) representative picture of G at day 15 stained with May–Grünwald Giemsa; (**L**) the graph represents the morphological analysis of G lineage differentiation/maturation; and (**I**,**J**,**L**) mean ± SEM of three independent experiments is shown. **Panel 4**. Growth and differentiation of MK unilineage culture: (**M**) cell growth analysis of MK unilineage culture generated by CB CD34^+^ HSCs; (**N**) flow cytometry analysis of CD34, CD41, CD42b, CD61 and CD62P expression during Mk differentiation; (**O**) representative picture of MK at day 15 stained with May–Grünwald Giemsa; (**P**) the graph represents the inspection of number of cells’ nuclei during megakaryocytic differentiation; and (**M**,**N**,**P**) mean ± SEM of three independent experiments is shown. Scale bar in panels C, G, K, O corresponds to 100 μm.

Figure 2 outlines the differentiation dynamics in low dimensional spaces, showing representative pictures of E, Mo, G and Mk precursors together with the marker expression (assessed by flow cytometry, as described in the figure legend) and morphological evaluation at different times of culture. This culture system allowed us to pinpoint progressive phases of hematopoietic differentiation/maturation and to analyze the expression of regulatory factors at discrete stages. Total RNA was extracted from CD34^+^ HSCs and from unilineage cultures of E, Mo, G and Mk progenitors/precursors at days 5, 10, and 15 (thereafter dubbed T1, T2, T3). Monocytic differentiation/maturation develops along approximately 20 days of unilineage culture. However, we chose day 15 as the last point of our analysis as, in subsequent stages of culture, monocytes undergo progressive differentiation into macrophages (Figure 2, panel 2). MiRNA profiles at each time point were generated with the miRXplore^®^ platform, while cytokine profiles were produced with the Luminex^®^ platform, as described in the Section 4.

**Figure 3:**
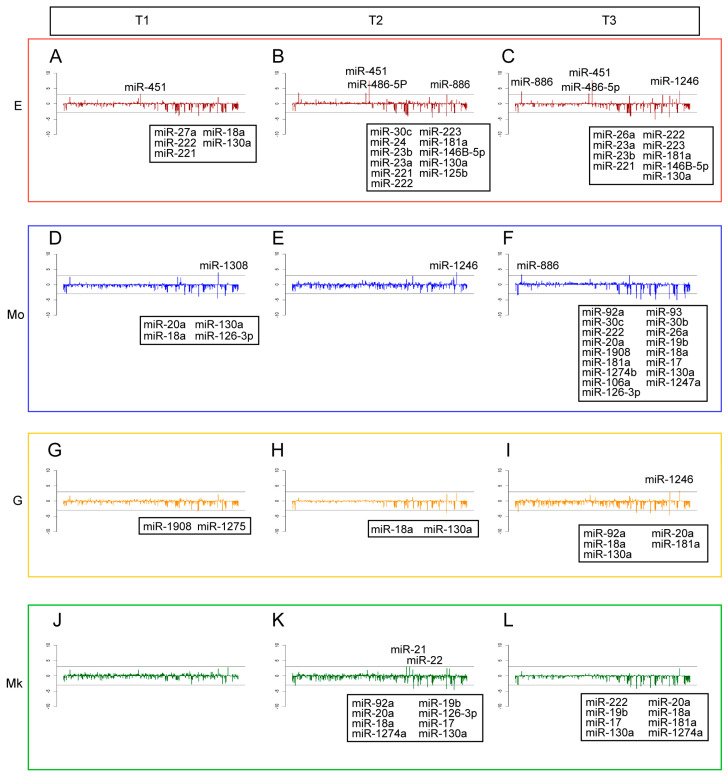
**Panel 1.** Barplot of logFC expression of miRNAs for E at time: (**A**) T1; (**B**) T2; (**C**) and T3. **Panel 2.** Barplot of logFC expression of miRNAs for Mo at time: (**D**) T1; (**E**) T2; and (**F**) T3. **Panel 3.** Barplot of logFC expression of miRNAs for G at time: (**G**) T1; (**H**) T2; and (**I**) T3. **Panel 4.** Barplot of logFC expression of miRNAs for Mk at time: (**J**) T1; (**K**) T2; and (**L**) T3.

**Figure 4:**
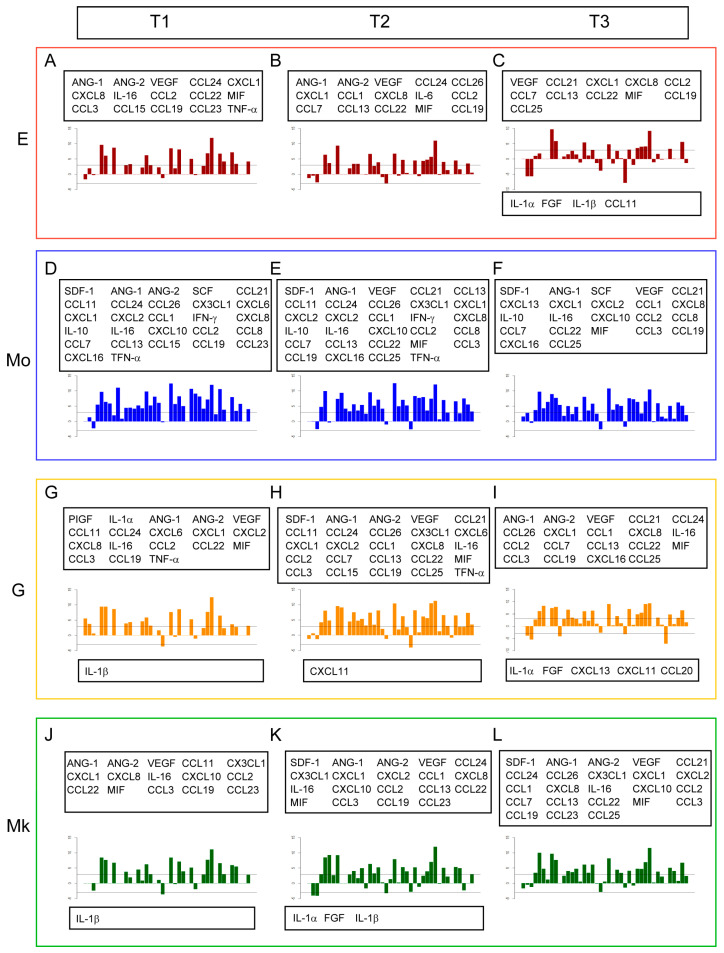
**Panel 1.** Barplot of logFC expression of cytokines for E in sequence at time: (**A**) T1; (**B**) T2; and (**C**) T3. **Panel 2.** Barplot of logFC expression of cytokines for Mo in sequence at time: (**D**) T1; (**E**) T2; and (**F**) T3. **Panel 3.** Barplot of logFC expression of cytokines for G in sequence at time: (**G**) T1; (**H**) T2; and (**I**) T3. **Panel 4.** Barplot of logFC expression of cytokines for Mk in sequence at time: (**J**) T1; (**K**) T2; and (**L**) T3.

The expression profiles of miRNAs and cytokines of the different lineages at separate times are shown in Figure 3 and Figure 4. For a better representation, we used log2 fold-change values calculated with respect to the common precursor CD34^+^ for miRNAs, and log2 transformation for cytokines. In both cases, the names of the miRNAs and cytokines that have an expression value ≥ |3| are reported. It is noteworthy that several of the microRNAs shown in Figure 3 have been experimentally validated and documented as being relevant in lineage-specific cell differentiation and maturation. For example, miR-451 regulates red blood cell production [12] and maturation by targeting GATA-2 [13] together with miR-486, which controls the erythroid versus megakaryocyte lineage fate decision and downregulates MAF [14]. MiR-22 was identified as a positive regulator of megakaryocyte differentiation by repressing GFI1 expression [15] together with miR-126, which plays a key role in platelet-supported thrombin generation. MiR-126 downregulates plexin B2 and enhances a PI3K/AKT signaling pathway by promoting calcium influx [16]. MiR-17 and miR-20a participate in the control of monocytopoiesis through AML1 targeting, M-CSF receptor upregulation [17], and by regulating the HIF transcription factor system [18]. The miR-181 family modulated granulocytic and macrophage-like differentiation by directly targeting PRKCD, CAMKK1 and CTDSPL mRNAs, and their expression is significantly increased in AML M1, M2 and M3 subtypes [19].

It is worth noting that most displacements from CD34^+^ stem cells are negative (decreasing concentration) for miRNAs (Figure 3) and positive (increasing concentration) for cytokines (Figure 4). This result is consistent with the prevailing negative correlations [20] between miRNAs and cytokines that was the basis for the choice of only taking into consideration miRNA–cytokine negative correlations in subsequent network analyses.

**Figure 5:**
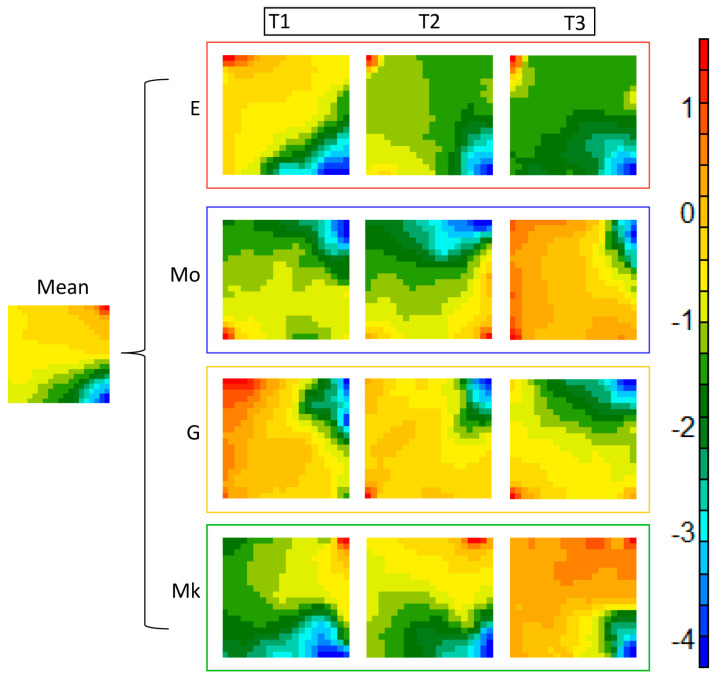
Self-organizing maps (SOM) used to show the development of hematopoietic lineages at various time intervals with two-dimensional representation. SOM projects the values of each miRNA (in terms of logFC with respect to CD34^+^ stem cells) on a bi-dimensional grid in which the values cluster according to a color scale (mean pattern on the left). Keeping invariant the mutual position of the miRNAs on the grid, new colors are assigned according to the actual values of each miRNA in the different conditions. This allows us to visually appreciate the different patterns of miRNAs of the lineages along the differentiation trajectory.

To obtain a visual summary of the different patterns of miRNA expression during the differentiation trajectories of the lineages, we adopted the so-called GEDI approach [21]. GEDI (Gene Expression Dynamics Inspector) is an application of self-organized maps (SOM) [22]. Each miRNA species corresponds to a node of a bi-dimensional network in such a way that the species with similar expression profiles are associated with the same region of the graph, creating areas of co-expressed miRNAs. Each lineage is projected on the map by means of a color code corresponding to its expression level on the reference lattice (mean) (corresponding to the average expression of each species across all the samples). Figure 5 reports SOMs in which the progressive change of the expression profile in time is clear, as well as the lineage specificity of the miRNA expression pattern. It is worth noting that the trajectories of the different lineages intersect, at various times, in accordance with the force-field character of the miRNA space, while at the same time showing some invariant features (i.e., the blue region on the upper right angle) blurring the line’s individuality.

The small number of cytokines makes a GEDI-like representation of this space irrelevant; thus, for cytokines, we preferred a simple correlation matrix heat-map.

To obtain a synthetic picture of differentiation taking explicitly into consideration the high dimensionality of the phenomenon, we adopted a simple correlation approach. We abandoned the idea of following each single miRNA (cytokine) trajectory, shifting to the analysis of the mutual resemblance of different line profiles in both miRNA and cytokine spaces. This is a common (and obligatory) choice when studying systems made by many interacting elements, like in the case of the correlations of amino acid residue trajectories in protein molecular dynamics [23].

**Figure 6:**
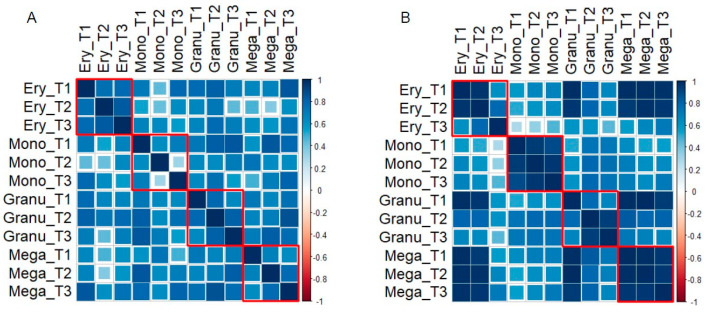
Correlations between samples in: (**A**) miRNA space; and (**B**) cytokine space.

Thus, we computed the pairwise Pearson *r* correlation between the different cell lines on both miRNA and cytokine spaces, obtaining the results reported in Figure 6.

As is evident in Figure 6, all profiles are positively correlated, pointing to a general resemblance across times and lines of both miRNA and cytokine average values. From a biological point of view, in addition to the global correlation, the recognition (darker squares corresponding to a higher correlation) of different lineages in the correlation matrix is relevant. This lineage-specific character of correlations (with near-to-unity values of Pearson *r*) is much more evident in the cytokine than in the miRNA spaces. This is proof of the more ‘stable’ character of state (cytokines) than the force-field (miRNA) space, according to our premises. 

This point is further confirmed by the average correlation values obtained within the lines (squares enclosed by red lines, see Figure 6). The numerical values of both within and between line correlations are reported in Table 1 and Table 2, respectively.

It is immediately noticeable how the line-specificity is higher for the cytokines than for miRNAs, as can be seen in the ratio within/between the lineages correlation that is equal to 0.87/0.80 = 1.09 for cytokines, and 0.69/0.78 = 0.88 for miRNAs.

To obtain a probabilistic appreciation of this result, we applied the *F*-test for the analysis of variance (ANOVA) to the correlation results to determine, by a two factors ANOVA, the non-random character of both individuality (within the lineage correlation) and miRNA–cytokine differences. As dependent variables, we used the correlation values obtained for miRNAs and cytokines. As independent variables, we used the factors ‘within/between lineages’ at two levels (Yes/No) and ‘miRNA/cytokine spaces’ at two levels (Yes/No). The results are shown in Table 3.

**Figure 7:**
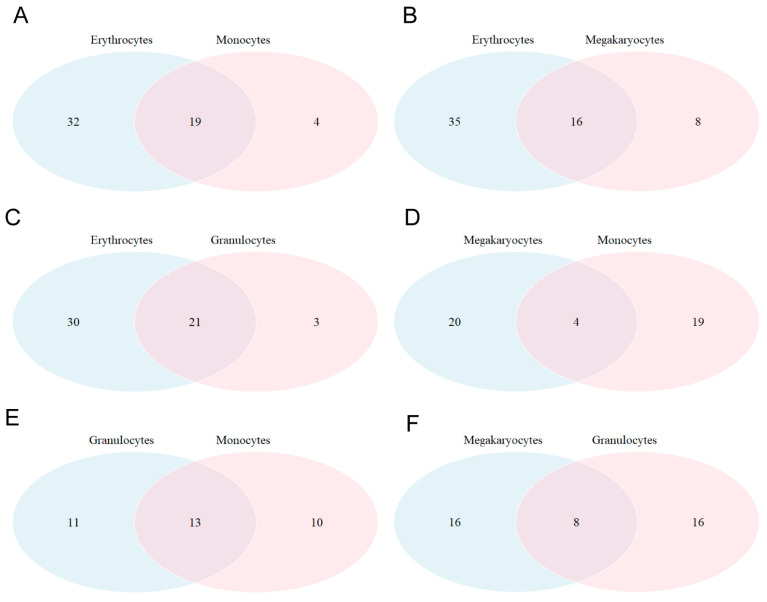
Venn diagrams highlight the number of shared miRNAs for various cell lines: (**A**) erythrocytes and monocytes; (**B**) erythrocytes and megakaryocytes; (**C**) erythrocytes and granulocytes; (**D**) megakaryocytes and monocytes; (**E**) granulocytes and monocytes; and (**F**) megakaryocytes and granulocytes.

The marked lineage-specific character of the differentiation process (prevalence of the internal correlation over the between-lineage correlation) is highly significant and goes hand in hand with a global increase in cytokine correlations in the miRNA space. These results are in close agreement with the ‘force-field’ and ‘state-space’ character of miRNAs and cytokines, respectively. Then, we selected the most significant miRNAs for each cell line by using an unsupervised k-means clustering procedure with a Euclidean metric (see Section 4). About a hundred miRNAs were obtained for the erythrocytes, while approximately seventy miRNAs were obtained for each of the remaining lineages. Through the Venn diagrams (shown in Figure 7), we present information on the number of significant miRNAs shared between the lineages and on those specific to each lineage. It also appears that there are around fifty miRNAs common to all cell lineages.

**Figure 8:**
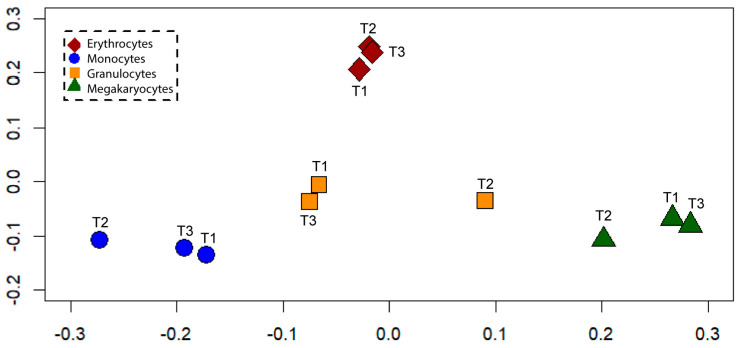
Metric multidimensional scaling with Jaccard distances, based on the presence/absence matrix for miRNAs. It shows the progressive differentiation of cell lineages from the common precursor CD34^+^.

So far, our analyses adopted a plain Euclidean metrics taking into consideration the actual numerical values of all the considered miRNA and cytokine species. These metrics, while being optimal for correlating miRNA and cytokine species, could severely bias the mutual distances between experimental samples. In fact, it underestimates the difference between the (very few) miRNA species, displaying a significant departure from the CD34^+^ ancestor and the bulk of species that are not significantly modified during differentiation trajectories. Furthermore, the presence of obvious ‘peaks’ emerging from miRNA profiles (see Figure 3) pushed us to adopt a Jaccard metrics [24] to obtain a synthetic bi-dimensional space to accommodate the different lineages and obtain a comprehensive explicit representation of their mutual relations (see Section 4). The adopted procedure takes into consideration only the miRNAs that emerged from the clustering analysis. The procedure for using the Jaccard distance consists of constructing a matrix that has all the initial miRNAs as statistical units and the various differentiation times of the cell lines as variables. A value of one is assigned for each significant miRNA in each line (even more than one) and a value of zero for the remainder. By doing so, we maintain ‘significant’ information, limiting the noise caused by miRNAs with weak or non-existent signals. The choice of Jaccard metrics is optimal in the case of sparse matrixes (see Section 4) in which the elevated proportion of zeros (miRNA species not significantly modified with respect to CD34^+^) heavily biases the usual Hamming distance metrics [25]. The multidimensional scaling applied to the between-samples Jaccard distances gave rise to the bi-dimensional plot reported in Figure 8, where the discrimination among different lineages is truly clear and fully concordant with their development path from the common precursor.

It is worth noting how the Jaccard metrics, relying only on the (small) fraction of miRNAs significantly different from the CD34^+^ state, allow us to single out the lineage specificity even in the miRNA space (that was barely present in the Euclidean space of the Pearson correlation). This corresponds to a sort of ‘derivative space’, considering as ‘active’ only the elements of the miRNome heavily modified with respect to the starting point (CD34^+^ profile). The conclusive step of the analysis was the modeling of the relations between the cytokine and miRNA spaces. Given the complexity of such interactions, we imposed some hard constraints on the analysis:

- Considering only the miRNA–cytokine pair correlations without considering the correlations inside each space;
- Limiting the analysis to significantly modified CD34^+^ miRNA species;
- Accepting only negative correlations (miRNAs exert direct inhibitory action on cytokines, the positive correlation being the consequence of indirect correlations stemming from the composition of two negative direct links);
- Limiting the analysis to the near-to-unity correlation (more negative than *r* = −0.9).

Network analysis was performed individually for each cell lineage, mapping only the miRNA/cytokine relationships that meet the previous constraints. For our purposes, it is useful to work on ‘communities’, defined as a subset of nodes within a graph, such that the connections between nodes are denser than the connections with the rest of the network. Community detection, as explained in [26], allows for cohesive and meaningful sub-graphs to be revealed that can allow for the features, functions, structure and dynamic of complex networks at the modular (community) level to be recognized.

Numerous algorithms aim to detect groups consisting of densely connected nodes with fewer connections between the groups; here, we relied on the Newman–Girvan algorithm (based on edge betweenness). The Newman–Girvan algorithm [27] is a hierarchical method for community structure detection and analysis based on the iterative elimination of edges that have the highest number of shortest paths between nodes passing through them. By removing edges from the graph one at a time, the network breaks down into smaller pieces, the so-called communities. The idea is to find which edges in a network occur most frequently between other pairs of nodes by finding the betweenness centrality of the edges. The edges joining the communities should, therefore, have high edge betweenness. The underlying community structure of the network will be much finer once the edges with the highest betweenness are eliminated (which means that communities will be much easier to detect). In addition, as network analysis provides deep insight into real complex systems, revealing the link between the topological and functional role of network elements can be crucial to understanding the mechanisms underlying the system [28]. Referring to the work of Guimerà and Amaral [29], we examined the role of nodes for each lineage, by calculating the within-module degree and the participation coefficient. In this way, we can classify nodes into universal roles according to their pattern of intra- and inter-module connections.

**Figure 9:**
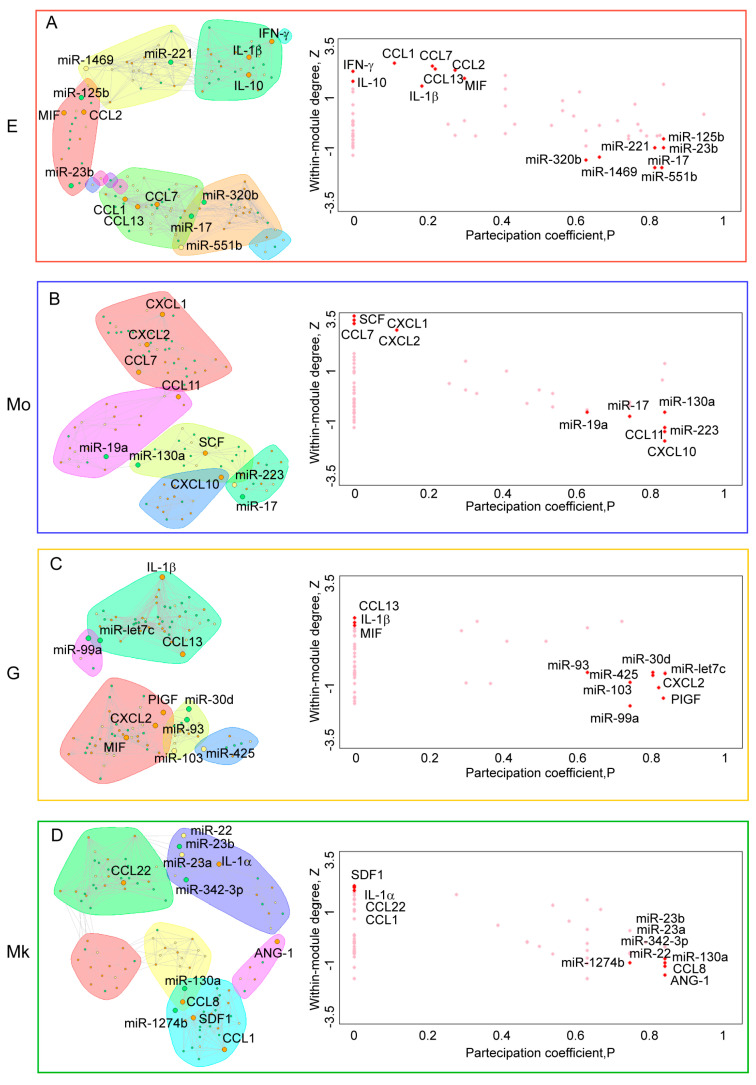
Network analysis with Newman–Girvan algorithm with associated *Z*–*P* plot for: (**A**) E lineage (modularity score = 0.64); (**B**) Mo lineage (modularity score = 0.63); (**C**) G lineage (modularity score = 0.53); and (**D**) Mk lineage (modularity score = 0.65). Different colors mark the different clusters (communities) emerging from Newman-Girman algorithm, the size of the colored areas are proportional to the number of elements of the corresponding cluster.

The within-module degree *Z_i_* measures how ‘well-connected’ node *i* is to other nodes in the module. High values of *Z_i_* indicate a high within-module degree, pointing to the central role of the node in organizing its specific module. The participation coefficient *P_i_* measures how ‘well-distributed’ the links of node *i* are among different modules. The participation coefficient *P_i_* is close to 1 if its links are uniformly distributed among all the modules, and 0 if all its links are within its own module (see Section 4). Our interest is aimed at the nodes that, in the two-dimensional *Z*–*P* representation, have high *Z* and low *P* (top left in the graph) and low *Z* and high *P* (bottom right in the graph). The first typology represents the nodes that have relevance only within the module they belong to, while the second represents the nodes that have the role of connector with different modules within the network. Furthermore, we computed the modularity index for each network to measure the strength of the division of a network into communities. Modular networks have dense connections between nodes within communities but sparse connections between nodes in different communities. Figure 9 reports all this information comprehensively for each cell lineage.

The analysis of both structural (high *Z* responsible for the modules’ resilience) and dynamical (high *P* favoring the variability of mutual arrangements between modules) [28] can potentially provide some hints on the nature of different lineage differentiations and functions. In addition to the modular organization level, it is worth investigating the general features of the miRNA–cytokine networks by means of the most common network descriptors, whose values are reported in Table 4.

It is worth noting the sparsity (low density/high diameter) of the monocytes network with respect to the other lineages corresponding to small communities. On the contrary, both granulocytes and megakaryocytes display a complex and intermingled regulation network corresponding to a more finely tuned phenotype. The nodes with high betweenness values in the various networks are identified. This is because, as highlighted in [30], a node with high betweenness is a node that is frequently on the shortest path between other nodes, thus mediating a relevant part of the information flux through the network. In other words, it acts as a ‘bridge’ between nodes within the network. This measure is useful for identifying key nodes that connect to other parts of the network. We then observe the relationships between these nodes, belonging to specific communities, and the cytokines occupying the more central (most-connected) location within them.

We obtained, for the miRNA/cytokine network relative to erythrocytes, the main relevance of miR-125b, miR-23b, miR-221, miR-17, miR-551B, miR-320b, miR-1469 and the cytokines IFN-γ, IL-10, CCL1, CCL7, CCL13, CCL2, MIF, IL-1β. For the miRNA/cytokine monocyte network, we obtained miR-19a, miR-17, miR-130a, miR-223, and the cytokines CCL11, SCF, CXCL10, CXCL1, CCL7, CXCL2.

Instead, for the miRNA/cytokine granulocyte network, we obtained miR-93, miR-103, miR-425, miR-30d, miR-99a, let-7c, and the cytokines CXCL2, PIGF, CCL13, MIF, IL-1β. For the megakaryocyte miRNA/cytokine network, the relevant role of miR-1274b, miR-23b, miR-23a, miR-342-3p, miR-22, miR-130a, and the cytokines SDF-1, IL-1α, CCL22, CCL1, CCL8 and ANG-1, was found.

In addition, the role of some nodes that simultaneously present high values for Z and *P* will be addressed in the discussion, as they play a key role in the connectivity of the networks. For erythroids, CCL8 and VEGF were found for monocytes miR-151 and IL-1α, for granulocytes CXCL11 and CXCL1, and for megakaryocytes miR-486, miR-1260 and CXCL11.

## 3. Discussion

The most common explanation style of a given cellular behavior, such as cell fate control, makes use of molecular pathways in which individual molecular regulatory events are linked by an ‘arrow-to-arrow’ scheme [31]. This pathway-based approach implies a linear chain of causation. Thus, cell behavior collapses to a sequence of deterministic causal relationships between symbols. This qualitative description is disconnected from the elementary principles of a dynamical system, which the cell’s regulatory network must obey [31]. Limiting ourselves to pathways analysis corresponds to discarding the basic fact that cell physiology is governed by a ‘self-sustaining’ intricate web of interactions living in a highly multidimensional state-space, whose energy minima mathematically correspond to a vector of concentration values of a huge number of molecules that define a stable cell type [31,32]. Pathways (when considered in isolation) derive from Newtonian dynamics characterized by a hierarchy of cause–effect relations. This style of reasoning implies a sort of ‘regressio ad infinitum’ toward an initial cause placed at the most basic organization layer. On the contrary, pathways are only partial views of a network system that in turn, at odds with Newtonian dynamics, implies a circular causality and asks for a different epistemology [32]. As aptly stressed by Donald Mickuleky in [32], these systems ask for a thermodynamic approach in which the central concept is the ‘state’. The state of a system cannot be defined in terms of microscopic causative chains (in the great majority of cases, the elemental players are too many and largely unknown) and its trajectory is driven by both internal and micro-environmental constraints to which the system adapts [31,32].

The case of blood cell differentiation is paradigmatic of this ‘thermodynamic’ approach; we have functionally well-defined trajectories unified by the same starting point (CD34^+^ HSCs) ending in diverse stable states (erythrocytes, monocytes, granulocytes, megakaryocytes) and acting as ‘attractors’ of the dynamical process. The choice of the specific fate of a cell is not strictly deterministic [33] but is driven by specific effectors imposing a preferential direction to the process [33].

As stressed above, the complete definition of the state of a cell is out of reach; however, we can select some ‘reduced’ versions of the complete phase space that offer a view of both the state and dynamics of the studied cell endowed with different ‘amounts of realism’. The only way to determine the relevance of a chosen state description emerges a posteriori by obtaining a statistically significant and stable discrimination among different cell fates in the chosen description space. This was the goal of this work that is demonstrated by both the scoring of widely different ‘within-lineage’ and ‘between-lineage’ correlations and by the possibility of projecting the different samples in a bi-dimensional space emerging from their different miRNA profiles (Figure 8).

Following the indications of a previous work by our group [7], we present an in-depth investigation into the character of the miRNome as a ‘force-field’, reporting on the transient regulatory state of the system, in contrast with a more ‘stable’ phenotypic description corresponding to the cytokine concentration profile. This working hypothesis was confirmed by the experimental results, demonstrating the stronger lineage discrimination ability of cytokines with respect to the miRNA space. In a previous study [7], the miRNome was contrasted with the transcriptome space that played the role of the ‘most stable’ description. Substituting the transcriptome with cytokines represents a drastic jump toward a more functionally relevant role played by miRNAs (cytokines space is a proper functionally interpretable description at odds with the transcriptome), while at the same time highlighting the possibility of obtaining an extremely robust description of the differentiation process. This result was (partially) unexpected; after all, mRNAs are the direct targets of miRNAs and the subsequent step to protein molecules concentrations (cytokines) is highly non-linear, with only weak expected correlations between mRNA and relative protein abundances at steady state [34]. This result, in naïve physical terms, corresponds to the lower level of correlations of a comfort zone (steady state) with respect to a rapidly changing condition (differentiation process) in the same manner as a set of independently wandering water molecules at steady state acquire a strong correlation (convective cells) only when moving toward a drastic phase transition (boiling point) [35,36].

The emergence of such strong correlations between miRNA and cytokine spaces prompted us to investigate, in greater detail, the character of this relation. Adopting a network formalism allows us to highlight the richness of mutual relations between the two spaces, moving beyond simple linear causative chains. The most relevant miRNA–cytokine interactions were the bases for generating interaction graphs, relative to the different lineages, that highlight a strong lineage dependency of miRNA–cytokine correlation patterns, together with the identification of the most important miRNA–cytokine interaction pairs at the basis of the blood cell differentiation process.

Figure 9 highlights a very important feature common to all the analyzed lineages: the high clustering coefficient (high-left quadrant of the scatter plots) zone contains only cytokine species, while the bottom-right quadrant corresponding to the high participation coefficient is prevalently made of miRNA species. This is consistent with the regulatory role in the development of miRNAs (force field) and the opposite phenotypic (state variables) role of cytokines. This implies that, while cytokines best mark the development phase of any lineage, the development dynamics are mirrored by miRNAs. This effect is present in any network system, as in the paradigmatic case of the allosteric effect in protein molecules [28].

From a more applicative point of view, we expect that eventual alterations of the high *P* nodes have a crucial role in terms of pathogenesis. This was exactly the case for the high *P* miRNAs we experimentally derived, namely: miR-125b, miR-22, miR-23a, miR-23b, miR-221, miR-223, miR-17, miR-551b, miR-320b, miR-1469, miR-19a, miR-130a, miR-93, miR-103, miR-425, miR-30d, miR-99a, let-7c, miR-1274b, and miR-342-3p. All these miRNAs were reported as being involved in normal/pathological hematopoiesis discrimination (see for example Refs. [37,38,39,40,41,42,43,44]). In rare cases (ANG-1, CCL8, PIGF, CXCL2, CXCL10, CCL11), cytokine species can have a high partition coefficient value. As we noted above for miRNAs, even in this case, nodes in charge of network long-range connections are involved in different pathologies (see for example Refs. [45,46,47,48,49,50,51]). Unlike the miRNAs shown in Figure 3, the role of some of these miRNAs during hematopoietic lineage-specific differentiation is currently unknown. Future studies will be required to elucidate the pathways regulated by these miRNA/cytokine networks.

In summary, our results highlight a previously unexplored interaction between miRNA and cytokine spaces during hematopoietic differentiation. The identification of miRNA networks of influence within the complex landscape of cellular and soluble factors that shape human hematopoiesis may contribute to future advancements in RNA-based therapies for hematologic disorders.

## 4. Materials and Methods

### 4.1. Human Cord Blood CD34^+^ HSCs Purification

Human cord blood (CB) was obtained, after informed consent, from healthy, full-term placentas according to the institutional guidelines of A.Fa.R. Centro Trasfusionale, Università La Sapienza, Rome, Italy and approved by the local ethical committees of Istituto Superiore di Sanità, Rome (file number # 171639). Isolation of CD34^+^ cells from CB, unilineage culture and morphological analyses were performed as described [52]. Briefly, low-density mononuclear cells were isolated and CD34^+^ cells were purified by positive selection using the MACS immunomagnetic separation system (Miltenyi Biotec, Bergisch Gladbach, Germany) according to the manufacturer’s instructions. The purity of CD34^+^ cells was assessed by flow cytometry using a monoclonal PE-conjugated anti-CD34 antibody (Miltenyi Biotec) and was routinely over 95% (range comprised between 92 and 98%). Each single experiment may have included pooled cells derived from different (2/3) cord blood.

### 4.2. Unilineage Differentiation

CD34^+^ progenitors were cultured in a BIT 9500 serum-free medium (STEMCELL Technologies Inc., Vancouver, BC, Canada) supplemented with human low-density lipoprotein (40 μg/mL) in the presence of various recombinant human cytokine combinations. For the erythroid unilineage culture, a serum-free medium was supplemented with 0.01 U/mL IL-3, 0.001 ng/mL GM-CSF (PeproTech Inc., Rocky Hill, NJ, USA) and 3 U/mL erythropoietin (Amgen, Thousand Oaks, CA, USA). For the megakaryocytic unilineage culture, the medium was supplemented with 100 ng/mL thrombopoietin (PeproTech). For granulocytic unilineage culture, the medium was supplemented with 1 U/mL IL3, 0.1 ng/mL GM-CSF combined with a plateau level of G-CSF (500 U/mL) (PeproTech). For the monocytic unilineage culture, the serum-free medium was supplemented with 1 ng/mL IL-6, 100 ng/mL Flt3-ligand, combined with plateau-level M-CSF (50 ng/mL) (PeproTech).

### 4.3. Cell Growth and Viability

Cells were cultured at 37 °C in a 5% CO_2_/5%O_2_/90%N_2_ atmosphere. Cell growth, the rate of proliferation, and the percentage of viable cells were analyzed by cell counting using Trypan Blue.

### 4.4. Morphology and Flow Cytometry Analysis

Cells were harvested on different days of the culture, smeared on slides by cytospin centrifugation, and stained with May–Grunwald Giemsa. A morphological analysis was performed to assess both cell maturation and the number of nuclear lobes per cell (400× magnification under a microscope, Eclipse 1000, Nikon, Tokyo, Japan, equipped with a digital camera). Analysis of the cell surface antigens was performed by flow cytometry using the following monoclonal antibodies (mAbs): fluorescein isothiocyanate (FITC) anti-CD15; phycoerythrin (PE); anti-CD11b; anti-CD14; anti-CD34; anti-CD41; anti-CD61; anti-CD62P; and anti-CD235a (GPA) (all from BD Pharmingen, San Diego, CA, USA). Phycoerythrin anti-CD42b (from Miltenyi Biotec, Bergisch Gladabach, Germany) was used. Briefly, the cells were suspended in Ca^2+^Mg^2+^ free phosphate-buffered saline solution (PBS) containing 20% FCS, mouse IgG (40 μg/mL), incubated for 10 min on ice and labelled with fluorochrome–conjugated mAbs for 30 min on ice. Cell fluorescence was analyzed with the FACS Canto (Becton Dickinson, Franklin Lakes, NJ, USA).

### 4.5. Cytokine Assays

The levels of 41 inflammatory and immune response mediators were evaluated by a combination of multiplex immune assays based on the Luminex technology (BioRad Hu chemokine custom 33-plex panel of a 40-plex panel #171AK99MR2 with the following analytes: CCL1/I-309, CCL2/MCP-1, CCL3/MIP-1α, CCL7/MCP-3, CCL8/MCP-2, CCL11/EOTAXIN, CCL13/MCP-4, CCL15/MIP-1δ, CCL19/MIP-3β, CCL20/MIP-3α, CCL21/6Ckine, CCL22/MDC, CCL23/MPIF-1, CCL24/EOTAXIN-2, CCL25/TECK, CCL26/EOTAXIN-3, CX3CL1/Fractalkine, CXCL1/Gro-α, CXCL2/Gro-β, CXCL6/GCP-2, CXCL8/IL-8, CXCL9/MIG, CXCL10/IP-10, CXCL11/I-TAC, CXCL12/SDF-1α+β, CXCL13/BCA-1, CXCL16/SCYB16, FGF basic/FGF2/bFGF, IFN-γ, IL-10, IL-16, IL-2, MIF, TNF-α, Bio-Rad, Hercules, CA, USA; Hu magnetic Luminex # LXSAHM-8 kit (Angiopoietin-1, Angiopoietin-2, IL-1α, SCF, VEGF, FGF basic, IL-1β, PIGF) and a Bio-Plex X200 System equipped with a magnetic workstation (Bio-Rad Laboratories, Hercules, CA, USA) according to the manufacturer’s instructions. The analysis was performed in duplicate. The Bio-Plex Manager Software 6.1 (Bio-Rad) assessed the quantification, and data were expressed as pg/mL [53].

### 4.6. RNA Extraction and Microarray Analysis

To examine microRNA expression in human hematopoietic cells, total RNA was extracted using TRIzol Reagent (Invitrogen Life Technologies, Carlsbad, CA, USA). The generation of miRNA profiles for T1–T3 of E, Mo, G and Mk cultures was conducted with the miRXplore microarray. The analysis of the miRNA microarray included procedures of background correction/normalization, re-ratio calculation/normalization, and dye-swap control experiments. Values relative to CD34^+^ HSCs were used to calculate the ratio between HSCs and each time point. This ratio was transformed using the log2 transformation to obtain the log2 fold change (logFC). A positive value indicates an upregulation of the miRNA compared to its control; a negative value indicates its downregulation.

### 4.7. Statistical and Bioinformatics Analysis

All statistical analyses were performed using R Statistical Software (version 4.4.0; R Core Team 2024). In the case of miRNAs, the data are expressed in terms of the log2 fold change (logFC) with respect to the common ancestor (CD34^+^), while the cytokines, defined by their concentration (pg/mL) in the different lineages, are normalized for subsequent analyses. Using the ‘kohonen’ package [54], a Gene Expression Dynamics Inspector (GEDI) analysis was applied to the scaled logFC values of microRNAs, creating heat-maps via the self-organizing maps (SOM) algorithm to group similarly behaving microRNAs. The selection of the most significant miRNAs was based on the results obtained from an unsupervised k-means clustering procedure with a Euclidean metric. A prerequisite for meaningful clustering is the coverage of at least 60% of time points with expression values for each miRNA. This analysis was carried out by Miltenyi Biotec, a German global biotechnology company. Through the ‘VennDiagram’ package [55], information was provided on the number of significant miRNAs shared by all the lines and those specific for each line.

The multidimensional scaling analysis (PCoA, principal correspondence analysis) of the miRNA space was derived from the Jaccard metrics defining the relative distance between *A* and *B* samples as follows:

$$D_{j}\left(A,B\right)=1-J\left(A,B\right)=1-\frac{\left(|A⋂B|\right)}{\left(|A\cup B|\right)}\;$$

Here, *A* and *B* correspond to two independent samples whose distance is a function of the number of shared significant miRNA species |*A∩B*| and of the species present in at least one of the two samples, with |*A*∪*B*| being *J*(*A*,*B*), the Jaccard similarity index. The presence/absence matrix stems from the relative superposition of miRNA species in the two samples. At odds with the miRNome, the cytokine patterns are expressed in terms of their actual concentration; this directed our choice toward a plain Euclidean space being any ‘presence/absence’-based metrics as arbitrary. To have a common basis to compute between profiles correlations in both spaces, we considered miRNA and cytokine concentrations as living in a Euclidean space in evaluating the pairwise Pearson correlation between samples with the consequent comparison of the line-discrimination power of the two spaces.

The complex network approach was performed with the ‘igraph’ package [56]. By adopting a Pearson correlation threshold *r* ≤ −0.9 for the insertion of an edge between two nodes (miRNA–cytokine), we generated the networks relating to the most significant interactions for the various lines, in terms of cell line-specific miRNAs and miRNAs common to all cell lines. Using the Newman–Girvan algorithm for identifying communalities and calculating the modularity index, networks were analyzed with respect to a series of indices that provided relevant information on their structural characteristics. In addition, for each node in the networks, we calculated the within-module degree *Z* index and the participation coefficient *p*, following the guidelines defined in [21]. The within-module degree index is as follows:

$$Z_{i}=\frac{K_{i}-{µ}_{K_{S_{i}}}}{\sigma_{K_{S_{i}}}}=\frac{A-B}{D},\;\;\;\;\;\;\;\;\;\;\;\;\left\{\begin{array}{l} \;A=K_{i}\\ \;B={µ}_{K_{S_{i}}}\\ \;C=S_{i}\\ \;D=\sigma_{K_{S_{i}}} \end{array}\right.$$

where *K* is the number of links of node *i* to other nodes in its module *A*, *B* is the average of *K* over all the nodes in *C* and *D* is the standard deviation of *K* in *C*, while the participation coefficient is as follows:

$$P_{i}=1-\sum_{s=1}^{N_{M}}{\left(\frac{K_{is}}{K_{i}}\right)}^{2}=1-\sum_{s=1}^{N_{M}}{\left(\frac{E}{F}\right)}^{2},\;\;\;\;\;\;\;\;\;\;\;\;\left\{\begin{array}{l} \;E=K_{is}\;\\ \;F=K_{i} \end{array}\right.$$

where *E* is the number of links of node *i* to nodes in module *s*, and *F* is the total degree of node *i*.

For each network, nodes that meet the conditions of a high *Z*-value and low *p*-value and low *Z*-value and high *p*-value were considered. Finally, their roles within the network in which they are located were discussed.

## Figures and Tables

**Table 1:** Within-lineage correlation for: (**A**) miRNA space (average within-lineage correlation = 0.69); and (**B**) cytokine space (average within-lineage correlation = 0.87).

A			B	
Lineage	Pearson *r*		Lineage	Pearson *r*
Erytrocytes	0.78		Erytrocytes	0.80
Monocytes	0.54		Monocytes	0.93
Granulocytes	0.74		Granulocytes	0.79
Megakaryocytes	0.70		Megakaryocytes	0.96

**Table 2:** Between-lineage correlation for: (**A**) miRNA space (average between-lineage correlation = 0.78); and (**B**) cytokine space (average between-lineage correlation = 0.80).

A				B		
Lineage 1	Lineage 2	Pearson *r*		Lineage 1	Lineage 2	Pearson *r*
Erytrocytes	Monocytes	0.66		Erytrocytes	Monocytes	0.85
Erytrocytes	Granulocytes	0.91		Erytrocytes	Granulocytes	0.67
Erytrocytes	Megakaryocytes	0.98		Erytrocytes	Megakaryocytes	0.92
Monocytes	Granulocytes	0.56		Monocytes	Granulocytes	0.95
Monocytes	Megakaryocytes	0.63		Monocytes	Megakaryocytes	0.82
Granulocytes	Megakaryocytes	0.96		Granulocytes	Megakaryocytes	0.62

**Table 3:** Result of *F*-test. The second and third columns show the value of the *F* statistic and the *p*-value, respectively.

Contrasting Factors	*F*-Value	Pr(>F)
within/between lineages	7.46	0.007
miRNA/cytokine spaces	5.69	0.01

**Table 4:** The following values are reported for each network: Edge density: the density of a graph is the ratio of the actual number of edges and the largest possible number of edges in the graph, assuming that no multi-edges are present; Diameter: a network diameter is the longest geodesic distance (length of the shortest path between two nodes) in the network; Centrality degree: the graph-level centrality index is based on degrees of vertices; Centrality closeness: the graph-level centrality index is based on the closeness of vertices; Centrality betweenness: the graph-level centrality index based on betweenness of vertices.

Lineage	Edge Density	Diameter	Centrality Degree	Centrality Closeness	Centrality Betweenness
Erytrocytes	0.07	16	0.14	0.09	0.49
Monocytes	0.05	20	0.23	0.08	0.44
Granulocytes	0.10	8	0.10	0.29	0.04
Megakaryocytes	0.07	13	0.13	0.07	0.14

## Data Availability

The original contributions presented in the study are included in the article/Supplementary Materials. Further inquiries can be directed to the corresponding authors.

## Supplementary Materials

The following supporting information can be downloaded at: https://www.mdpi.com/article/10.3390/ijms252212305/s1.
